# Effect of the Nutritional Intervention Program on Body Weight and Selected Cardiometabolic Factors in Children and Adolescents with Excess Body Weight and Dyslipidemia: Study Protocol and Baseline Data

**DOI:** 10.3390/nu15163646

**Published:** 2023-08-19

**Authors:** Beata Bondyra-Wiśniewska, Anna Harton

**Affiliations:** Department of Dietetics, Institute of Human Nutrition Sciences, Warsaw University of Life Sciences (WULS), 159C Nowoursynowska Str., 02-776 Warsaw, Poland

**Keywords:** childhood obesity, adolescents, excess body weight, BMI, dyslipidemia, intervention program, low-glycemic index diet

## Abstract

Excess body weight and associated dyslipidemia in children and adolescents are the main risk factors for cardiovascular diseases in young adults. There is a reasonable need to develop an effective lifestyle modification program that includes various dietary therapies. A low-glycemic index (GI) diet may be recommended in the treatment of obesity. Its use is also recognized as reasonable in cardiovascular diseases, including dyslipidemia. The aim of the presented nutritional intervention program was to evaluate the effectiveness of an energy-balanced diet based on the principal recommendation on Cardiovascular Health Integrated Lifestyle Diet-2 (CHILD-2) and low-GI products (LGI diet) in children and adolescents with excess body weight and dyslipidemia. The study involved 64 children and adolescents (44 boys and 20 girls) aged 8–16 with overweight or obesity and dyslipidemia. For 8 weeks, the participants followed a dietary treatment using two types of diets: one based on products with a low GI, and one standard therapy diet. During this time, they participated in three visits with a dietitian, during which the assessment of their current and habitual food intake was made, and anthropometric measurements and blood pressure were taken. Patients were under the care of a pediatrician who qualified them for the study and ordered lipid profile tests. This article presents the design, protocol of the nutritional intervention program, and baseline data. The collected results will be used to develop practical nutritional recommendations for children and adolescents with excess body weight and dyslipidemia.

## 1. Introduction

Childhood obesity is one of the fastest-spreading civilization diseases of the 21st century. According to World Health Organization (WHO) data [1], the prevalence of overweight and obesity among children and adolescents aged 5–19 has increased significantly, from 4% in 1975 to just over 18% in 2016. Lifestyle changes over the last few decades are mainly responsible for such a high prevalence of excess body weight in children and adolescents [2,3]. Among them, the most important are unhealthy diet, low physical activity, and sedentary lifestyle (e.g., in front of a computer screen, TV). Global estimates of scientists indicate that, if appropriate preventive measures are not taken, by 2025, about 268 million children and adolescents aged 5–17 will be overweight, 91 million of them obese [4]. This worrying trend has also been observed in Poland. Results of the Health Behavior in School-Aged Children (HBSC) study show that over the years 2002–2014, the percentage of Polish teenagers with obesity aged 11–15 doubled [5]. It is estimated that in Poland, 18.2% of teenagers aged 13–19 have excess body weight, of which 11.6% are overweight and 6.6% are obese [6].

The rapidly progressing prevalence of overweight and obesity in children and adolescents results in health consequences. The most common are disorders of lipid metabolism, which occur in 17% to even 74% of children and adolescents with excess body weight, depending on the studied population [7]. These are risk factors for cardiovascular diseases at a young age. Obesity and dyslipidemia in children have been shown to be associated with increased carotid intima-media thickness, which increases the risk of premature atherosclerosis [8,9]. Lowering the age limit for the occurrence of lipid disorders increases the risk of cardiovascular complications (including myocardial infarction, heart failure, and stroke) in young adults who had excess body weight in childhood [10]. The analysis of data from four longitudinal cohort studies including adolescents aged 12–18 showed that a higher risk of atherosclerosis in adulthood was associated with the presence of overweight (2 times higher risk), obesity (3.7), borderline high low-density lipoprotein cholesterol (LDL-C) (1.6), and borderline low high-density lipoprotein cholesterol (HDL-C) (1.4) in teenagers [11]. A meta-analysis of 21 studies showed a significant association between childhood obesity and high triglyceride (TG) levels and lower HDL-C levels in adulthood [12]. All this contributes to the deterioration in the quality and shortening of life and increases the cost of treatment. Therefore, early treatment of obesity and lipid disorders in children and adolescents is important, which may prevent the development of cardiovascular and other diet-related diseases in the future.

There is a reasonable need to develop an effective lifestyle modification program aimed at the growing group of children and adolescents with overweight/obesity and dyslipidemia, including various dietary therapies [13]. It is known that in the case of adults, a low-glycemic index (GI) diet can be effective both in reducing body weight and improving lipid parameters [14,15]. However, for children and adolescents, data on the effectiveness of this type of diet are still lacking [16]. A well-planned diet therapy program should be comprehensive and based on appropriate and current recommendations, which have not been updated in the last few years, either in Poland or the rest of the world [17,18]. In therapy, multidisciplinary care should be provided—its effectiveness in reducing body weight is greater with the care of a dietitian [7]. The decrease in body mass index (BMI) was associated with the improvement in lipid parameters. The purpose of the program was to evaluate the effectiveness of an energy-balanced diet based on the principal recommendation on Cardiovascular Health Integrated Lifestyle Diet-2 (CHILD-2) [17], and additionally on low-GI products (LGI diet) in children and adolescents with excess body weight and dyslipidemia.

### Study Aims and Hypothesis

The main purpose of this study was to evaluate the effect of a nutritional intervention program on weight loss and the improvement in lipid parameters in children and adolescents with excess body weight and dyslipidemia. The secondary aims of the study were to assess the intervention’s effect on body composition, waist and hip circumference, and blood pressure. The nutritional intervention program included two types of diets: (1) based on low-GI products (LGI diet)—intervention group, and (2) a standard therapy (ST diet)—control group. Both diets were based on the principal recommendation of the Cardiovascular Health Integrated Lifestyle Diet-2 (CHILD-2) [17]. A detailed description of the dietary procedure is included in the methodology.

Research hypotheses:

(a) The nutritional intervention program will induce a reduction in body weight and improve lipid parameters within 8 weeks of the recommended dietotherapy;
(b) The LGI diet may be a more effective form of dietotherapy compared to the ST diet.

The nutritional intervention program should initiate a change in eating habits for further weight loss benefits. Our goal is to translate research results into guidelines useful in designing a multidisciplinary program for children and adolescents with overweight or obesity and dyslipidemia that is effective in reducing body weight and improving lipid parameters. The primary aim of this article is to describe the design and the study protocol of the nutritional intervention program. The secondary purpose is to present the baseline characteristics of the study group.

## 2. Methods

### 2.1. Study Design

**Figure 1:**
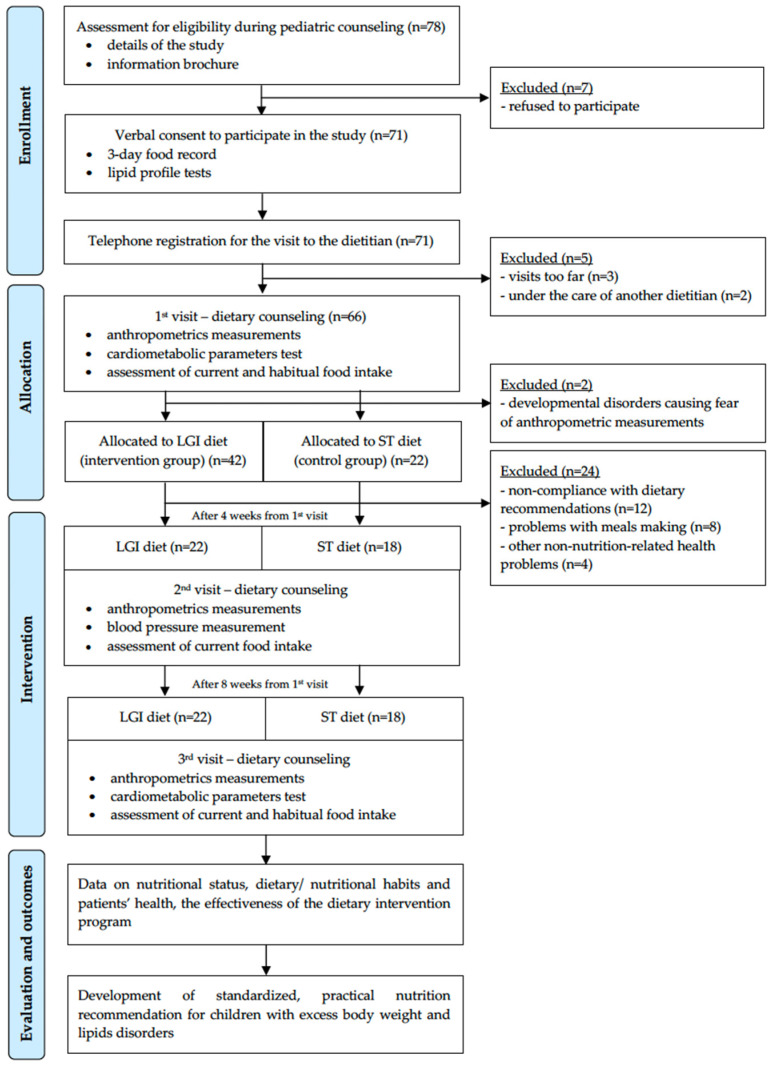
The nutritional intervention program—study design.

The study design is presented in Figure 1. This program is an intervention study that was conducted from 2019 to 2020. Within 8 weeks, participants and their parents/primary caregivers attended 3 visits with a dietitian at 4-week intervals. Patients were randomly assigned to one of two groups: the intervention (LGI diet) or control group (ST diet).

The study protocol was approved by the Ethics Committee of the Faculty of Human Nutrition and Consumer Science, Warsaw University of Life Sciences WULS, Poland (10p/2017, 17 May 2017).

### 2.2. Participants and Sample Estimation

Children and adolescents with overweight or obesity and dyslipidemia were qualified for this study by a pediatrician from The Children’s Memorial Health Institute in Warsaw (Poland) based on a medical interview. In total, 64 of 78 eligible children and parents/primary caregivers agreed to participate in this study (44 boys and 20 girls aged 8–16 years) (Figure 1).

The estimated minimum sample size takes into account the following parameters:

- Size of the general population: The number of children aged 7–18 living in the Mazowieckie Voivodship with overweight/obesity—these were the initial assumptions and criteria for inclusion in the study (*n* = 140,211).
- Significance level (0.05) and confidence level of 95%, maximum estimation error (5%).
- In the estimates of the sample size, the assumption of repeatability of the examined feature was adopted, i.e., the size of the fraction at the level of 0.039 (3.9%—prevalence of lipid disorders in the population of children in Poland).

Minimum sample size was performed for a finite population. The estimated sample size is 58.

### 2.3. Eligibility Criteria

Criteria for participation included: (1) age 7 to 18 years old, (2) residence in Poland in the Mazowieckie Voivodeship, (3) a BMI ≥ 85th percentile for age and gender based on the Polish growth reference values as defined by International Obesity Task Force (IOTF) [19,20], and (4) dyslipidemia coexisting with excess body weight. Dyslipidemia is the presence of at least 1 lipid abnormality, such as high total cholesterol (TC), high low-density lipoprotein (LDL-C), high triglycerides (TG), or low high-density lipoprotein cholesterol (HDL-C). The participants did not use any pharmaceuticals, dietary supplements, or nutraceuticals affecting changes in the parameters of the lipid profile and blood pressure or supporting weight loss.

The study exclusion criteria were: (1) age below 7 or above 18 years, (2) BMI < 85th percentile for age and gender based on the Polish growth reference values as defined by IOTF [19,20] and BMI > 35 kg/m^2^, (3) lipid abnormality resulting from genetic diseases (e.g., congenital dyslipidemias, type I diabetes), (4) the use of pharmaceuticals that affect changes in the parameters of the lipid profile or blood pressure, (5) chronic diseases (due to the possibility of using pharmacotherapy), (6) having metal implants (e.g., metal sutures), (7) having devices that send an electrical signal (e.g., a pacemaker or heart defibrillator), and (8) having epilepsy. The last three exclusion criteria are a contraindication to body composition analysis [21,22].

### 2.4. Dietary Procedure

The patients were randomly assigned to the intervention group (LGI diet) [17,23,24,25] or the control group (ST diet) [17]. Both diets were based on the main principles of the Cardiovascular Health Integrated Lifestyle Diet-2 (CHILD-2) recommendations. The energy value of the diets was individually adjusted to the degree of excess body weight [26]. The energy value of the diets was based on the basal metabolic rate (determined using the analyzer body composition TANITA MC-780 P MA) and level of physical activity [27]. The diets were matched for macronutrient composition: 15–20% from protein, 25–30% from fats (saturated fatty acids < 7%), and 50–60% from carbohydrates for daily energy. Energy distribution was 20–25% at breakfast, 15–20% at 2nd breakfast, 35–40% at lunch, 5–10% at afternoon snack, and 10–15% at supper.

The main assumption of the LGI diet was to consume products with a low GI (<55) [28] such as whole-grain products, low-starch vegetables, and raw fruits, with a reduced content of simple sugars, nuts, and seeds [17,25]. On the other hand, eliminating processed products, which are a source of sugars and salt, and limiting the consumption of saturated fatty acids and replacing them with unsaturated fatty acids, were recommended in both diets [17]. When planning both diets, the recommendations of the Polish Pyramid of Healthy Nutrition and Lifestyle for Children and Adolescents [29] were additionally considered.

Each patient received a diet plan that assumed the consumption of 5 meals a day with instructions for its use. Each diet plan included 10 dishes for each meal with similar energy and nutritional value. Patients were free to choose their preferred dish at each meal. The description of the dishes included their name, the list of ingredients necessary for their preparation, amounts of ingredients expressed in home measures (e.g., spoon, glass) and grams, and the method of preparation.

### 2.5. General Procedure

#### 2.5.1. Enrollment

The nutritional intervention program’s study design is presented in Figure 1. During the medical visit, the pediatrician qualified patients for the program and explained the details of the study to the patient and the parent/primary caregiver. They then received a brochure with information about the study, including the aim, duration and procedure of the program, inclusion and exclusion criteria, how to prepare for a visit to the dietitian due to the body composition analysis performed, possible side effects resulting from dietary changes (mainly due to the more fiber consumed), place of visits, and contact details of the dietitian. Patients also received a 3-day food record template with instructions on how to complete it. The pediatrician ordered tests for TC, LDL-C, HDL-C, and TG in the fasting blood sample. The doctor interpreted the test results, made a diagnosis of a lipid disorder, and recorded the results in the patient’s medical chart [30]. Patients were then enrolled for the 1st visit and test results were reported to the dietitian.

Before each visit to the dietitian, parents/primary caregivers and children were informed about how to prepare the child for body composition analysis [21,22,31]. Measurements were taken under fasting conditions or at least 4 h after a meal and at least 12 h after vigorous exercise. Additionally, participants were instructed to avoid consuming caffeinated drinks (e.g., coffee, energy drinks), use the toilet immediately before the measurement, and remove jewelry and any metal elements (e.g., belt).

#### 2.5.2. First Visit—Baseline

At the first visit, the dietitian again explained the aim and procedure of the study. Written consent for the child’s participation in the study was obtained from parents/primary caregivers and adolescents over 13 years old. Data on children were collected such as age, birth weight, time spent in moderate-to-vigorous physical activity, and screen time [32]. Selected sociodemographic data were collected on the parent/primary caregiver who was involved in nutritional counseling and childcare during the study (see Appendix A).

The dietitian analyzed and discussed participants’ current food intake based on the 3-day food record and analyzed the lipid parameters results provided by the pediatrician. The dietitian then collected habitual food intake data and performed anthropometric measurements (i.e., heigh, weight, waist, hip and arm circumference, body composition) according to the Anthropometry Procedures Manual by the National Health and Nutrition Examination Survey (NHANES) [33]. Table 1 presents a description of the parameters and measurement methods used. Based on weight and height, the body mass index (BMI) was calculated using the following formula: BMI [kg/m^2^] = weight [kg]/(heigh [m])^2^. BMI was compared with the Polish growth reference values [19]. Overweight was defined as a BMI between 85th and 95th percentile, and obesity as > 95th percentile for age and gender, as defined by IOTF [20]. BMI reference values for healthy children and adolescents range from the 5th to the 85th percentile.

Patients were randomly assigned to the intervention group (LGI diet) or the control group (ST diet). Each of them received a diet plan individually adjusted to their energy needs, which is described in detail in Section 2.4. Dietary procedure. All subjects were asked to record all meals, drinks, and dietary supplements consumed during their participation in the program. On this basis, adherence to the dietary treatment was assessed and all nutrient calculations were performed using a table of nutritional value of food products and dishes [37]. Patients received initial nutrition education and had time to ask questions. At the end of the visit, they were also informed about the possibility of contacting the dietitian by phone or e-mail throughout their participation in the program.

#### 2.5.3. Second Visit—After 4 Weeks

After 4 weeks of following the diet plan, a second visit to the dietitian took place. The current food intake was analyzed and discussed, and anthropometric and blood pressure measurements were taken, as described in Figure 1 and Table 1. Participants had time to ask questions and share insights on the eating plan they were following.

#### 2.5.4. Third Visit—After 8 Weeks

As before, the dietitian assessed and discussed the current food intake and performed anthropometric as well as blood pressure measurements. Information on habitual food intake and lipid profile was also collected (Figure 1 and Table 1). At this last visit, the participants and their parents/primary caregivers received the necessary information and educational materials to help them correctly compose their meals after completing the study. Patients are under the care of a dietitian and a pediatrician (visits every six months).

## 3. Statistical Analysis

All statistical analyses were conducted using Statistica version 13.1 (Copyright©StatSoft, Inc., 1984–2014, Cracow, Poland). For all tests, *p* < 0.05 was considered significant.

The Mann–Whitney U test was used to compare the quantitative results due to the lack of normal distribution in the groups. The chi-squared test was used to examine the relationship between individual percentages in the case of qualitative data. Each superscript letter represents a subset of categories in different groups whose column proportions are not significantly different from each other. Two of the same letters mean no significant difference, and two different letters mean a significant difference in the result.

## 4. Key Findings at the Beginning of the Study

The basal characteristics of the study group are presented in Table 2 and the selected characteristic distribution of all participants is presented in Table 3. A total of 64 participants took part in the study (44 boys and 20 girls). There were significantly more boys in the LGI diet than in the ST diet group (*p* = 0.004). In the LGI diet group, statistically higher parameter values were noted for WHR (*p* < 0.001), percentage of body water (*p* = 0.004), and percentage of skeletal muscle mass (*p* = 0.007) compared to the ST diet group. In turn, the percentage of body fat was significantly higher in the ST diet group (*p* = 0.004). In other anthropometric parameters, there were no significant differences between the two groups before starting the nutritional intervention. Most of the study group spend less time on daily physical activity than the 60 min recommended by WHO (72%), and have more than 2 h a day of screen time (69%). Average screen time in the ST diet group was significantly longer compared to the LGI diet group, by about 73 min (*p* = 0.004).

The characteristics of the cardiometabolic parameters for all participants are presented in Table 4, and the baseline selected distribution of cardiometabolic parameters is presented in Table 5. There were no significant differences between the LGI diet and ST diet groups in all mean values for cardiometabolic parameters. Similarly, for most cardiometabolic parameters, there were no significant differences in the distribution of participants between groups. Only in the case of LDL-C there were significantly more participants with borderline high cholesterol in the ST diet group compared to the LGI diet group (*p* = 0.012). The most common lipid abnormality in the group was high triglyceride levels—none of the participants had an acceptable level of triglycerides.

## 5. Discussion

There is a great need to find practical and effective methods of dealing with the challenges of excess body weight and its health consequences in children and adolescents [40]. This article provides a rationale for the nutritional intervention program for children and adolescents with overweight or obesity and dyslipidemia. The strength of this study is a comprehensive approach to the care of a pediatric patient, including the care of a pediatrician and a dietitian [7]. Another advantage of this study is the use of a dietary intervention involving two types of diets—one based on low-GI products [23,24,25], and one standard therapy diet—both based on the principal recommendations of CHILD-2 [17]. In the available literature, there are data confirming the effectiveness of a standard therapeutic diet in reducing body weight in children and adolescents with overweight/obesity [41,42]. On the other hand, a low-glycemic index diet is not a typical approach to weight loss and dyslipidemia treatment. Therefore, the available literature still lacks new data on the impact of the GI on weight reduction in children and adolescents with excess body weight and lipid disorders. The LGI diet specifically focuses on carbohydrate quality products (LGI products), which affects glycemia and is related to body weight. It was observed that the excess consumption of simple sugars may be associated with excess body weight [43,44] and risk factors for cardiovascular diseases [44,45,46]. This is also indicated in the WHO recommendations (2015), also addressed to healthy people, both children and adults [47].

An important aspect of the presented program was the individual approach to the patient; the use of a diet consisting of interchangeable meals with similar energy and nutritional values made it easier to adjust the diet to the individual preferences of patients. It is also valuable that this program additionally qualified individuals for the presence of at least one lipid abnormality diagnosed by a pediatrician. Lipid disorders are a significant problem among children and adolescents with obesity [7]. Lipid disorders can occur even in overweight patients; therefore, patients with overweight should be provided with specialist care, which is why these patients were included in this study.

The added value of the study is the comprehensiveness of the program—many parameters were assessed (including anthropometric and cardiometabolic values, and current and habitual food intake), which can be used as a measure of the effectiveness of the therapeutic process [48]. This study will provide new insights into the possible health effects of LGI and ST diets on participants’ health. The results of the program will be used to develop standardized, practical nutritional recommendations for children with excess body weight and dyslipidemia. The discussed issues may serve as a guideline for those conducting similar trials with children and adolescents with overweight/obesity and dyslipidemia.

## Figures and Tables

**Table 1:** Description of parameters and applied measurement methods.

Parameter	Method of Measurement
**Anthropometrics**	
Height	No shoes, headgear, or head ornaments. Measured twice with a height meter to the nearest 1 mm. If there were any differences, the results were averaged. Height was compared with the Polish growth reference values [19].
Weight	In light clothes, no shoes, and heavy items in pockets (e.g., phone, wallet). Measurement taken using the professional TANITA MC-780 P MA multi-frequency body composition analyzer with weighing function to the nearest 100 g. Weight was compared with the Polish growth reference values [19].
Waist circumference	Waist circumference was measured in a standing position. Body weight was evenly distributed over both feet. After a few natural breaths, with freely relaxed abdominal muscles, waist circumference was assessed at the midpoint between the lower costal margin and the iliac crest at the end of a normal expiration. Measured twice with an anthropometric tape to the nearest 1 mm. If there were any differences, the results were averaged. Waist circumferences were compared with the Polish reference values [34]. Reference values for healthy children and adolescents: <90th percentile.
Hip circumference	Hip circumference was measured in a standing position. Body weight was evenly distributed over both feet. Hip circumference was assessed around the widest part of the buttocks. Measured twice with an anthropometric tape to the nearest 1 mm. If there were any differences, the results were averaged. Hip circumferences were compared with the Polish reference values [34]. Reference values for healthy children and adolescents: <90th percentile.
Arm circumference	Arm circumference was measured on a freely lowered non-dominant arm, with relaxed muscles. Arm circumference was assessed at the midpoint between the tips of the shoulder and elbow, in the place where the arm circumference is greatest. Measured twice with an anthropometric tape to the nearest 1 mm. If there were any differences, the results were averaged. Arm circumferences were compared with the percentile reference ranges [35]. Reference values for healthy children and adolescents: <90th percentile.
Body composition analysis	Body composition analysis was performed in a standing position. Body weight was evenly distributed over both feet. In light clothes, with no shoes, socks, tights, or heavy items in pockets (e.g., phone, wallet). Measurement taken using the professional TANITA MC-780 P MA multi-frequency body composition analyzer. Before each measurement, the electrodes were thoroughly wiped with an appropriate disinfectant.
**Cardiometabolics**	
Lipid profile (including levels of TC, HDL-C, LDL-C, and TG)	Tests performed after referral and under the supervision of the physician by qualified medical personnel in the laboratory. Lipid profile values were compared by pediatrician with the reference ranges according to the American College of Cardiology [30]. Acceptable values for healthy children and adolescents: <170 mg/dL for TC, >45 mg/dL for HDL-C, <110 mg/dL for LDL-C, <75 mg/dL for TG in children aged 0–9 years, and <90 mg/dL for TG in children and adolescents aged 10–19 years.
Blood pressure	The measurement was taken using an automatic upper-arm blood pressure monitor intended for children and adolescents. The cuff was placed on the left arm at the level of the heart, with the arm resting on the tabletop, the back resting on the back of the chair, and the feet resting on the floor. The measurement was taken in a sitting position after min, with 10 min of rest, twice at approximately 5 min intervals. Blood pressures were compared with the Polish reference values [36]. Systolic and diastolic blood pressure reference values for healthy children and adolescents: <90th percentile.
**Food intake**	
Current food intake	Current food intake was assessed using a 3-day food record before the 1st visit and each day throughout the duration of the diet intervention. Calculations of energy value and all nutrients from the food records were made by the dietitian with the use of a table of nutritional value of food products and dishes [37].
Habitual food intake	Habitual food intake was assessed using the validated Food Frequency Questionnaire (FFQ-6) [38]. FFQ-6 is used to collect information on the frequency of consumption of 62 assortment groups of products, representing 8 main food groups: (1) sweets and snacks, (2) dairy products and eggs, (3) grain products, (4) fats, (5) fruits, (6) vegetables, legumes, and nuts, (7) meat and fish products, and (8) drinks.

TC—total cholesterol; HDL-C—high-density lipoprotein cholesterol; LDL-C—low-density lipoprotein cholesterol; TG—triglyceride.

**Table 2:** Characteristics of the study group before the start of the intervention (mean ± SD).

Variable	Total (*n* = 64)	LGI Diet (*n* = 42)	ST Diet (*n* = 22)	*p*-Value (Mann–Whitney U Test)
Age [years]	12.78 ± 2.65	12.33 ± 2.73	13.64 ± 2.32	ns
Birth weight [g]	3355.94 ± 387.22	3398.10 ± 394.91	3275.45 ± 367.39	ns
Moderate or high-intensity physical activity [min/day]	40.12 ± 38.43	40.83 ± 36.91	38.77 ± 43.04	ns
Screen time [min/day]	172.77 ± 90.17	147.55 ± 82.46	220.91 ± 86.13	0.004
Anthropometrics				
Height (cm)	164.86 ± 16.17	164.17 ± 17.09	166.19 ± 14.53	ns
Body weight (kg)	75.66 ± 25.46	75.13 ± 28.32	76.67 ± 19.39	ns
Body weight-for-age percentile	94.39 ± 5.87	94.45 ± 5.55	94.26 ± 6.57	ns
BMI (kg/m^2^)	26.94 ± 5.23	26.75 ± 5.74	27.29 ± 4.16	ns
BMI-for-age percentile	94.46 ± 4.60	94.32 ± 4.70	94.72 ± 4.51	ns
Arm circumference (cm)	29.96 ± 4.38	29.83 ± 4.51	30.19 ± 4.51	ns
Waist circumference (cm)	94.00 ± 16.79	95.22 ± 17.83	91.67 ± 14.72	ns
Hip circumference (cm)	101.85 ± 13.26	99.81 ± 13.57	105.75 ± 11.99	ns
WHtR	0.57 ± 0.06	0.58 ± 0.06	0.55 ± 0.07	ns
WHR	0.92 ± 0.08	0.95 ± 0.07	0.86 ± 0.08	<0.001
FM (kg)	25.04 ± 11.56	24.55 ± 13.22	25.99 ± 7.61	ns
Percent of body fat (%)	32.11 ± 4.72	31.32 ± 5.06	33.61 ± 3.67	0.004
FFM (kg)	50.62 ± 15.09	50.58 ± 16.42	50.69 ± 12.50	ns
TBW (kg)	37.06 ± 11.05	37.03 ± 12.03	37.10 ± 9.16	ns
Percent of TBW (%)	49.68 ± 3.46	50.27 ± 3.70	48.57 ± 2.69	0.004
MM (kg)	48.04 ± 14.39	48.02 ± 15.67	48.09 ± 11.92	ns
SMM (kg)	28.61 ± 8.55	28.61 ± 9.31	28.61 ± 7.05	ns
Percent of SMM (%)	38.41 ± 2.62	38.81 ± 2.80	37.64 ± 2.06	0.007

BMI—body mass index; WHtR—waist to height ratio; WHR—waist to hip ratio; FM—fat mass; FFM—free fat mass; TBW—total body water; MM—muscle mass; SMM—skeletal muscle mass; ns—not significant; LGI diet—low-glycemic index diet; ST diet—standard therapy diet.

**Table 3:** Baseline selected characteristic distribution of all participants (*n*/% of participants).

Variable		Total (*n* = 64)	LGI Diet (*n* = 42)	ST Diet (*n* = 22)	*p*-Value (Chi-Squared Test)
Gender	Male	44/69	34 ^a^/81	10 ^b^/46	0.004
	Female	20/31	8/19	12/54	
Birth weight percentile	<90	56/88	36/86	20/91	ns
	≥90	8/12	6/14	2/9	
BMI interpretation	Overweight	28/44	20/48	8/36	ns
	Obesity	36/56	22/52	14/64	
WC percentile	<90	6/9	2/5	4/18	ns
	≥90	58/91	40/95	18/82	
Moderate or high-intensity physical activity—minimum 60 min a day as recommended by WHO [32]	Yes	18/28	14/33	4/18	ns
	No	46/72	28/67	18/82	
Screen time (hours/day) [39]	<2	20/31	16/38	4/18	ns
	≥2	44/69	26/62	18/82	
Parent’s level of education	Higher	34/53	24/57	10/46	ns
	Secondary	20/31	14/33	6/27	
	Vocational	10/16	4/10	6/27	
Financial situation	Very good	6/9	2/5	4/18	ns
	Rather good	40/63	24/57	16/73	
	Average	18/28	16/38	2/9	
Place of living	Village	20/31	10/24	10/46	ns
	Town < 100,000 citizens	20/31	14/33	6/27	
	Town ≥ 100,000 citizens	24/38	18/43	6/27	

BMI—body mass index; WC—waist circumference; WHO—World Health Organization; ns—not significant; LGI diet—low-glycemic index diet; ST diet—standard therapy diet; ^a,b^ statistically significant differences between LGI and ST diets.

**Table 4:** Characteristics of cardiometabolic parameters for all participants before the start of the intervention (mean ± SD).

Variable	Total (*n* = 64)	LGI Diet (*n* = 42)	ST Diet (*n* = 22)	*p*-Value (Mann–Whitney U Test)
SBP (mmHg)	118.22 ± 8.54	117.38 ± 6.99	119.82 ± 10.92	ns
SBP-for-age percentile	70.16 ± 22.44	69.52 ± 19.91	71.36 ± 27.11	ns
DBP (mmHg)	71.13 ± 5.38	71.90 ± 4.00	69.64 ± 7.21	ns
DBP-for-age percentile	80.94 ± 18.65	85.29 ± 9.73	72.64 ± 27.36	ns
Heart rate (bpm)	73.59 ± 5.85	73.95 ± 6.37	72.91 ± 4.75	ns
TC (mg/dL)	204.05 ± 41.94	203.60 ± 43.41	204.90 ± 39.96	ns
HDL-C (mg/dL)	42.25 ± 12.54	43.30 ± 14.67	40.24 ± 6.71	ns
LDL-C (mg/dL)	112.26 ± 19.23	113.05 ± 21.33	110.74 ± 14.76	ns
TG (mg/dL)	224.15 ± 112.10	208.38 ± 107.90	254.25 ± 116.29	ns

SBP—systolic blood pressure; DBP—diastolic blood pressure; TC—total cholesterol; HDL-C—high-density lipoprotein cholesterol; LDL-C—low-density lipoprotein cholesterol; TG—triglyceride; ns—not significant; LGI diet—low-glycemic index diet; ST diet—standard therapy diet.

**Table 5:** Baseline selected distribution of cardiometabolic parameters for all participants (*n*/% of participants).

Variable		Total (*n* = 64)	LGI Diet (*n* = 42)	ST Diet (*n* = 22)	*p*-Value (Chi-Squared Test)
SBP-for-age percentile	<90	48/75	32/76	16/73	ns
	≥90	16/25	10/24	6/27	
DBP-for-age percentile	<90	42/66	24/57	18/82	ns
	≥90	22/34	18/43	4/18	
TC	Acceptable	14/22	10/24	4/18	ns
	Borderline high *	24/37	14/33	10/46	
	High *	26/41	18/43	8/36	
HDL-C	Acceptable	12/19	8/19	4/18	ns
	Borderline high	20/31	14/33	6/27	
	High	32/50	20/48	12/55	
LDL-C	Acceptable	38/60	30 ^a^/71	8 ^a^/36	0.012
	Borderline high	20/31	8 ^a^/19	12 ^b^/55	
	High	6/9	4 ^a^/9	2 ^a^/9	
TG	Acceptable	0/0.0	0/0	0/0	ns
	Borderline high	8/12	8/19	0/0	
	High	56/88	34/81	22/100	

SBP—systolic blood pressure; DBP—diastolic blood pressure; TC—total cholesterol; HDL-C—high-density lipoprotein cholesterol; LDL-C—low-density lipoprotein cholesterol; TG—triglyceride; ns—not significant; LGI diet—low-glycemic index diet; ST diet—standard therapy diet; ^a,b^ statistically significant differences between LGI and ST diets. * The cut points for borderline high and high lipid values represent approximately the 75th and 95th percentiles, respectively [30].

## Data Availability

The datasets used and/or analyzed during the current study are available from the corresponding author upon reasonable request.
